# Neurocognitive function declines are reversible following migraine headache in college students

**DOI:** 10.1186/1129-2377-14-S1-P74

**Published:** 2013-02-21

**Authors:** MT Moore, T Covassin, KA Pfeiffer, RE Norris, RL Jensen, CF Branta

**Affiliations:** 1 Northern Michigan University, USA; 2 Michigan State University, USA

## Introduction

Computerized testing of neurocognitive function yields an accurate and reliable assessment [1]. There is little research on short-term effects of migraine headaches on neurocognitive function or their cognitive recovery patterns[2].

## Purpose/background/objective

The purpose of this study was to investigate neurocognitive function and recovery patterns in college students who incur migraine headaches compared to college students who do not.

## Methods

Volunteers (ages 18-29) completed computerized neurocognitive baseline (B) testing. Forty-four migraineurs incurring a migraine (M) were matched to 44 non-migraine (NM) controls for sex, age and education level. Verbal and visual memory, processing speed and reaction time were measured at 24 hours, 48 hours and 7 days post migraine.

## Results

Repeated measures ANOVAs revealed declines in neurocognitive function of migraineurs in verbal memory [mean diff(md)(24hr-B) M=-1.59±7.82,NM=1.19±7.69; =.045], visual memory [md (24hr-B)M=-4.70 +15.61, NM=3.05+10.94; p=.041), and reaction time [md(24hr-B)M=.02±.09, NM=-.01±.04.
